# Optimal duration of Vitamin K antagonists anticoagulant therapy after venous thromboembolism: a systematic review and network meta-analysis of randomized controlled trials

**DOI:** 10.1186/s12872-020-01345-z

**Published:** 2020-02-03

**Authors:** Wei Wang, Yang Su, Chunyan Wu, Yuxi Sun, Neng Dai, Wei Chen, Jie Zhang, Yawei Xu, Ralph G. Brindis, Dachun Xu, Jue Li

**Affiliations:** 1 Key Laboratory of Arrhythmias of the Ministry of Education, Shanghai East Hospital, Tongji University School of Medicine, Tongji University, Shanghai, 200120 People’s Republic of China; 2 Institute of Clinical Epidemiology and Evidence-based Medicine, Tongji University School of Medicine, 1239 Siping Road, Shanghai, 200092 China; 3 Department of Cardiology, Shanghai Tenth People’s Hospital, Tongji University School of Medicine, 1239 Siping Road, Shanghai, 200092 China; 4 Department of Medicine & the Philip R. Lee Institute for Health Policy Studies, University of California, San Francisco, CA USA

**Keywords:** Anticoagulants, Hemorrhage, Recurrence, Meta-analysis, Venous thromboembolism

## Abstract

**Background:**

The optimal duration of oral anticoagulant therapy for patients with venous thromboembolism (VTE) remains highly uncertain in clinical practice. It is essential to accurately assess the effect of anticoagulant therapy in reducing recurrent VTE against the risk of inducing major bleeding.

**Methods:**

Randomized controlled trials were identified by searching PubMed, Web of Science, Embase, and the Cochrane library, reporting rates of recurrent VTE and major bleeding in patients taking Vitamin K Antagonists (VKA) with VTE and comparing different durations.

**Results:**

Eleven RCTs with 3109 participants utilizing varied durations were included in the meta-analysis. Longer VKA therapy was associated with significantly lower rates of VTE recurrence compared with shorter duration of VKA therapy (OR 0.75, 95%CI 0.57–0.99), with significant difference noted in major bleeding risk (OR 2.31, 95%CI 1.17–4.56). During anticoagulation duration, patients treated by 6-month VKA had higher risk of major bleeding compared with 3-month VKA regimen (OR 33.45, 95%CI 2.00–559.67).

**Conclusions:**

Regimen longer than 6 months did not show statistical elevation of major bleeding risk. VKA treatment strongly reduces the risk of recurrent VTE during anticoagulation therapy. The absolute risk of recurrent VTE declines over time while the risk for major bleeding after 6 months’ treatment did not demonstrate a continuous significant increase with extended duration of VKA therapy.

## Background

Venous thromboembolism (VTE) is a common disorder, which encompasses deep vein thrombosis (DVT) and pulmonary embolism (PE). VTE is regarded as the third major cause of cardiovascular disease after myocardial infarction and stroke [1]. VTE frequently occurred in two or three per 1000 persons per year [2]. Anticoagulant therapy has been widely used for the treatment for VTE for decades [3]. Vitamin K antagonists (VKAs) (i.e. warfarin, acenocoumarol, fluindione) have been the mainstay of long-term anticoagulant therapy. Warfarin, the most common VKA, remains the primary approach for which efficacy is well established. Although emerging alternatives utilizing the novel oral anticoagulants for VTE have now been investigated in some large randomized controlled trials, the cost advantages of VKA cannot be ignored, especially among low and middle-income countries. The low price of warfarin makes VKA therapy more available for use in larger patient populations at risk for VTE [4]. Warfarin is administered and its dose adjusted with regular laboratory monitoring while its anticoagulant effect can be reversed by Vitamin K given orally or intravenously and/or with fresh frozen plasma.

Bleeding is the most important complication associated with patients receiving oral anticoagulants. Previous studies demonstrate that the overall incidence of bleeding events in patients treated with long-term anticoagulation is 2 to 3% per year [5]. The duration of anticoagulation therapy is determined by the risk of VTE recurrence balanced by the risk of anticoagulant related bleeding events [6].

The Tenth edition of the American College of Chest Physicians Guidelines updated its recommendation that patients with either a first or a second VTE should receive extended treatment if their bleeding risk is low or moderate [3]. The safety paradigm should be taken into consideration when the treatment duration is prolonged. Several systematic reviews have investigated the effectiveness of different durations of treatment but none have concomitantly focused on the aspects of bleeding risk [6].

For patients with a first unprovoked VTE, the optimal duration of anticoagulation is a crucial clinical dilemma that has yet to be fully resolved. The decision to stop anticoagulant therapy after the initial 3–6 months or to continue anticoagulation therapy indefinitely, is primarily governed by the long-term risk of recurrence after treatment is discontinued. In this manuscript we performed this network meta-analysis of randomized controlled trials (RCTs) enrolling patients with deep vein thrombosis (DVT) or pulmonary embolism (PE) to compare the relationship of bleeding and the duration of VKA therapy.

## Methods

### Study design and identification

Eligible studies included in this meta-analysis were randomized clinical trials comparing different durations of anticoagulation in patients with venous thromboembolism. We systematically searched PUBMED (from inception to August, 2018), Web of Science, Embase, and Cochrane Library of clinical trials database (from 1972 to August, 2018) to identify randomized clinical trials (RCTs) without restrictions on the publication year, or type of publication [7]. The search was restricted to articles pertaining to adult humans and published in English language or translated into English. The following search terms were used: “warfarin”, “Vitamin K Antagonist”, “venous thromboembolism”, “pulmonary embolism”, “deep vein thrombosis”, “duration”, “months”, “random” and “randomized controlled trial”.

Two investigators (WW, CW) completed the systematic search and reviewed relevant articles according to the following criteria: 1) RCTs involving consecutive patients with venous thromboembolism of more than 50 participants who were assigned to receive VKAs; 2) the diagnose of venous thromboembolism was confirmed by either positive Doppler ultrasonography, radioisotope, venography, angiographic, or other objective tests; 3) studies comparing different durations of anticoagulant therapy in each article sorted by four categories: 3 months, 6 months, 12 months and longer than 24 months; 4) and studies reporting the clinical incidence of recurrent VTE events and major bleeding episodes. Observational studies, trials of shorter than 3 months or less than 50 participants, trials of non-VKA agents, studies in special population (e.g., patients with cancer), and studies with no available thromboembolic events or major bleeding data were excluded.

### Outcomes

The primary efficacy clinical outcome of interest was incidence of recurrent VTE and the primary adverse event clinical outcome measure was major bleeding episodes. Recurrent VTE was diagnosed if the symptoms were confirmed by ultrasonography, ventilation-perfusion lung scanning, spiral computerized tomographic angiography, pulmonary angiography, or autopsy.

We define major bleeding as clinically overt bleeding associated with 1 or more of the requirements as follows: bleeding occurred in a critical organ (intracranial, gastrointestinal, intraocular, retroperitoneal, or intraspinal); required transfusion of 2 or more units of whole blood or packed cells; fetal bleeding; and any reasons leading to interruption of anticoagulation treatment.

The definitions of clinical outcomes in each original article were integrated and compiled. Analyses were done in the intention-to-treat population. Secondary endpoints included recurrent VTE and major bleeding episodes which occurred during the period of anticoagulation and from discontinuation of VKA therapy to the end of follow-up.

### Data extraction and quality assessment

Data for eligible study were extracted by two investigators (WW, CW) independently including study design, patient characteristics, outcomes and other relevant information. All extracted data were reviewed by the same two reviewers and discrepancies were resolved through consensus discussion. The risk of bias of each trial were assessed by two review authors according to the Cochrane Risk of Bias assessment tool which comprise the following domains: random sequence generation; allocation concealment; blinding of participants and personnel; blinding of outcome assessment; incomplete outcome data; selective reporting and other bias, and were checked by other two reviewers (Dr Jue Li and Dr. Dachun Xu). Each domain was given a score of “high,” “low,” or “unclear” [8].

### Data synthesis and statistical analysis

We performed this meta-analysis using both a traditional frequentist approach and a frequentist network meta-analysis method. Our first analysis was based on the comparison between groups of therapies stratified as “longer duration” versus “shorter duration” of anticoagulation using Stata 14.0 (StataCorp LP, College Station, TX). For indirect treatment estimates, frequentist network meta-analysis was then conducted to synthesize both direct and indirect evidence to provide the most comprehensive results by means of R software by use of *netmeta* (R package (version)). The odds ratios (ORs) were reported the dichotomous outcomes (recurrent VTE and major bleeding episodes). The pooled estimates were calculated with either fixed effect (Mantel-Haenszel) or random effect (DerSimonian and Laird) models when direct meta-analysis was performed.

In the light of different length of follow-up of each trial, the event rates were arithmetically generated as estimates per patient-years of follow-up. For subgroup analyses, we categorized recurrent and major bleeding events both in the period of taking VKA and from discontinuation to the end of follow-up. Heterogeneity was assessed using I^2^ statistic across studies, with values higher than 50% representing significant heterogeneity, 25–50% indicating moderate heterogeneity, lower than 25% denoting mild heterogeneity [9]. Studies were merged by a fixed-effect model using Mantel-Haenszel procedure if there is no significant heterogeneity, otherwise a random-effect model were applied according to the approach of DerSimonian and Laird.

Ranking probabilities for efficacy and safety on different durations were presented using rankograms as their surface under the cumulative ranking (SUCRA) curves to obtain the treatment hierarchy [10]. Small study effects and publication bias were assessed by funnel plots and Egger’s test. Visual inspection was performed to examine funnel-plot symmetry, with *P* value less than 0.05 for Egger’s test suggesting publication bias or small study effect. Additionally, we conducted a sensitivity analysis to evaluate the strength of our findings based on removing particular trials.

## Results

Overall, a total of 11 RCTs comprising a total of 3109 patients with VTE who met the inclusion criteria in the meta-analysis (Additional file 3: Figure S1); baseline characteristics of these studies are described in Additional file 1: Table S1. Our design was limited to those studies with population who had objectively confirmed VTE. The sample size ranged from 64 to 749 participants, with a median sample size of 283. The anticoagulation duration between longer and shorter arms of enrolled studies ranged from 3 months to indefinite duration, with the median follow-up period of 33.3 months (range 12–48 months). The involved studies including 11 two-group trials assessing four VKA regimens: warfarin therapy (6 trials [11–16]), fluindione (1 trial [17]), warfarin plus acenocumarol (3 trials [18–20]), and warfarin plus dicumarol (1 trial [21]). Four RCTs evaluated a 3-month versus a 6-month of VKA therapy. Three RCTs assessed a 3-month versus 12-month VKA regimen. One trial [19] both evaluated 3 months versus either 6 months or 12 months VKA treatment. Four RCTs evaluated extended VKA therapy (no shorter than 24 months) compared with a 6-month regimen, while only 1 RCT assessed the comparison of extended treatment with a 3-month regimen. The network diagram of all the eligible treatment comparisons is illustrated in Additional file 4: Figure S2 in the supplement.

### Entire period of VKA anticoagulation

**Fig. 1:**
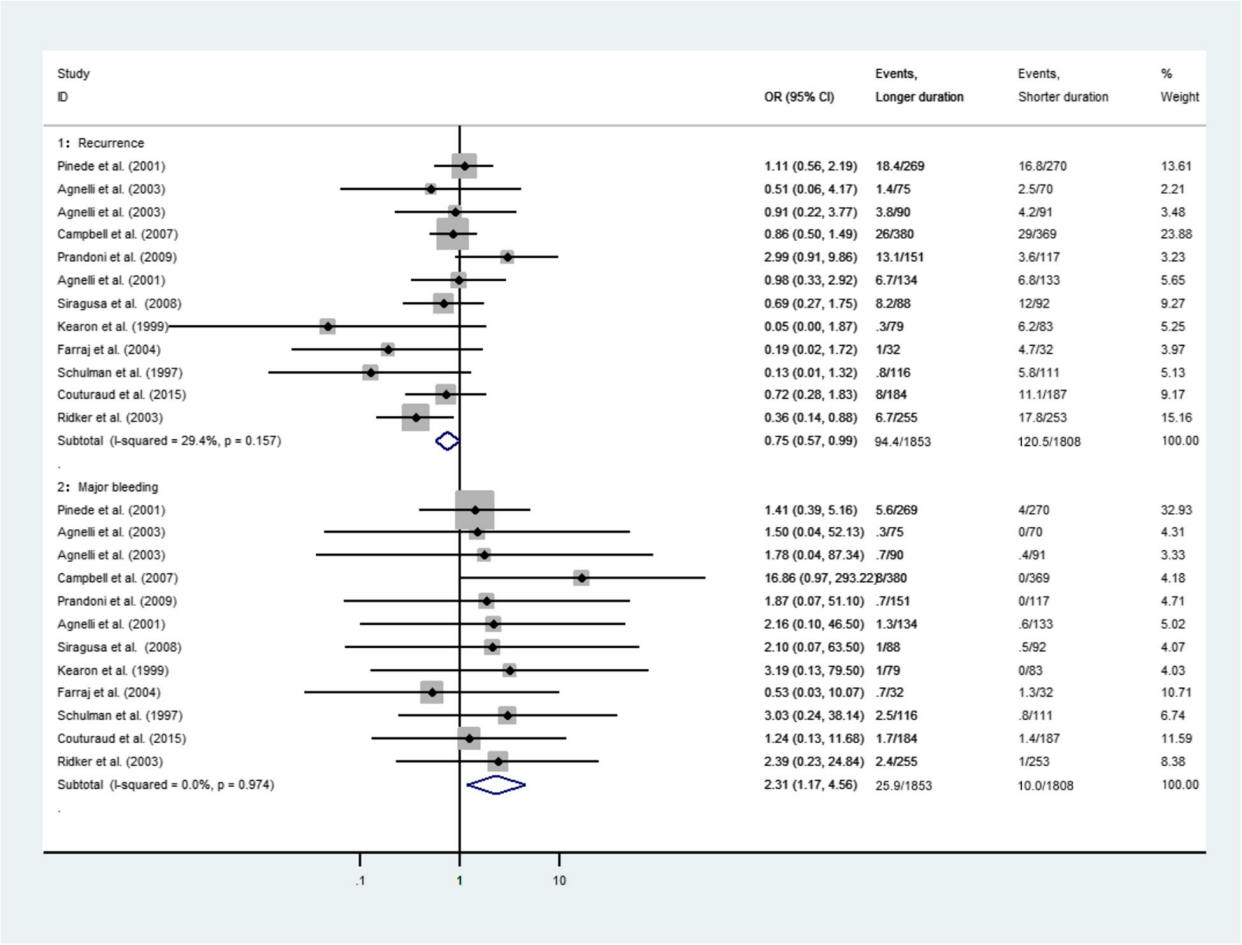
Pooled risk for VTE recurrence and major bleeding between longer and shorter duration of anticoagulation of the entire period

All the included trials reported data on recurrent VTE and major bleeding episodes. Figure 1 shows estimates of risk of recurrent VTE and major bleeding between longer and shorter VKA anticoagulation in the traditional meta-analysis. Longer duration VKA therapy was associated with significantly lower rates of VTE recurrence compared with shorter arms (OR 0.75, 95%CI 0.57–0.99), while significant difference was noted in major bleeding risk (OR 2.31, 95%CI 1.17–4.56). Moderate heterogeneity was recorded in trials for recurrent VTE (I^2^ = 29.4%) and no heterogeneity was apparent for major bleeding risk (I^2^ = 0).

In the frequentist network meta-analysis approach, it suggested that taking VKA over 24 months and 6-month regimen were associated with a significantly lower risk of recurrent VTE (OR 0.41, 95%CI 0.20–0.84 and OR 0.40, 95%CI 0.22–0.73, respectively) when compared with 3-month regimen, while there were no significant differences for major bleeding risk among four durations (Table 1). However, there was strong increasing trend for major bleeding risk when duration was extended. The probability analysis shows that taking VKA no shorter than 24 months had the lowest risk of VTE recurrence (P_best_ 94.2%, SUCRA 0.979). Nevertheless, a 3-month duration was ranked as the safest regimen relating to major bleeding, followed by 12-month, 6-month and 24-month therapy (P_best_ 70.0%, SUCRA 0.899) (Additional file 5: Figure S3). No heterogeneity was obvious across trials for recurrent or major bleeding (I^2^ = 0) in the network meta-analysis. No funnel plot asymmetry was noticeable by visual inspection and the Egger test suggesting no excessive effect exerted by small studies on the overall results (Additional file 6: Figure S4).

**Table 1:** Network-meta analysis estimates of entire period treatment duration for VTE patients

	Odds ratio (95% Confidence Interval) for outcome of major bleeding			
Odds ratio (95% Confidence Interval) for outcome of VTE recurrence	A (3 months)	2.05 (0.75, 5.66)	2.03 (0.28, 14.58)	3.25 (0.76, 13.91)
	**1.03 (0.69, 1.53)**	B (6 months)	0.99 (0.11, 9.07)	1.58 (0.49, 5.07)
	**0.82 (0.43, 1.55)**	**0.80 (0.38, 1.68)**	C (12 months)	1.60 (0.14, 18.43)
	**0.41 (0.20, 0.84)**^**a**^	**0.40 (0.22, 0.73)**^**a**^	**0.51 (0.19, 1.31)**	D (≥24 months)

The entire period results from network meta-analysis are presented as odds ratio (95% Confidence Intervals) between the column-defining and row-defining treatment duration. Odds ratio for comparisons are in the cell in common between the column-defining and row-defining treatment. For outcome of VTE recurrence, row treatment is compared with column treatment (ie, column treatment is reference). For outcome of major bleeding, column treatment is compared with row treatment (ie, row treatment is reference). Numbers in brackets indicate 95% Confidence Intervals. Numbers in bold represent statistically significant results. Data in bold represents the primary efficacy outcome (VTE recurrence) and the rest represents the primary safety outcome (major bleeding)

*VTE* venous thromboembolism

A-D: Treatment duration

^a^ Statistically significant results

#### Subgroup analysis (During anticoagulation and from discontinuation to the end of follow-up)

Additional file 7: Figure S5A-B shows subgroup analyses by traditional meta-analysis for each outcome in those trials which data could be derived. For the period of anticoagulation, 4 studies were incorporated for assessing recurrence of VTE, and 3 studies for evaluating major bleeding (Additional file 7: Figure S5A). With traditional meta-analysis, longer duration of VKA therapy did not achieve significant reduction for recurrent VTE (OR 1.00, 95%CI 0.61–1.64) compared with shorter regimens. There was also not any significant difference observed for safety outcome (OR 1.97, 95%CI 0.13–30.02). However, severe heterogeneity for major bleeding (I^2^ = 77.0%) was evident across trials, thus a random effect model was applied. Frequentist network meta-analysis revealed that there were no statistically differences among four VKA regimens, whereas 6-month therapy showed an increased risk of major bleeding (OR 33.45, 95%CI 2.00–559.67) when compared with 3-month (Additional file 2: Table S2). Nevertheless, similar result was not observed in 24-month regimen.

With regard to the period of after discontinuation of VKA, efficacy and safety outcomes were reported in 7 studies and 6 studies separately (Additional file 7: Figure S5B). Traditional meta-analysis suggested that no significant differences in VTE recurrence was recorded between longer and shorter duration of VKA therapy when anticoagulation stopped but longer regimen offered a protection (OR 0.19, 95%CI 0.04–0.88). Similarly, frequentist network meta-analysis also demonstrated longer therapy did not reduce recurrent VTE episodes statistically after discontinuation of VKA. While compared with 3-month regimen, we noted that 6-month treatment has the highest risk of major bleeding (OR 33.45, 95%CI 2.00–559.67) by network meta-analysis (Additional file 2: Table S2). Longer regimen was associated with a trend towards lower risk of major bleeding after anticoagulation. All network meta-analysis was performed in the fixed effect model considering that no heterogeneity was indicated (I^2^ = 0). Further, there were no inconsistencies between direct and indirect comparisons.

### Sensitivity analysis

**Fig. 2:**
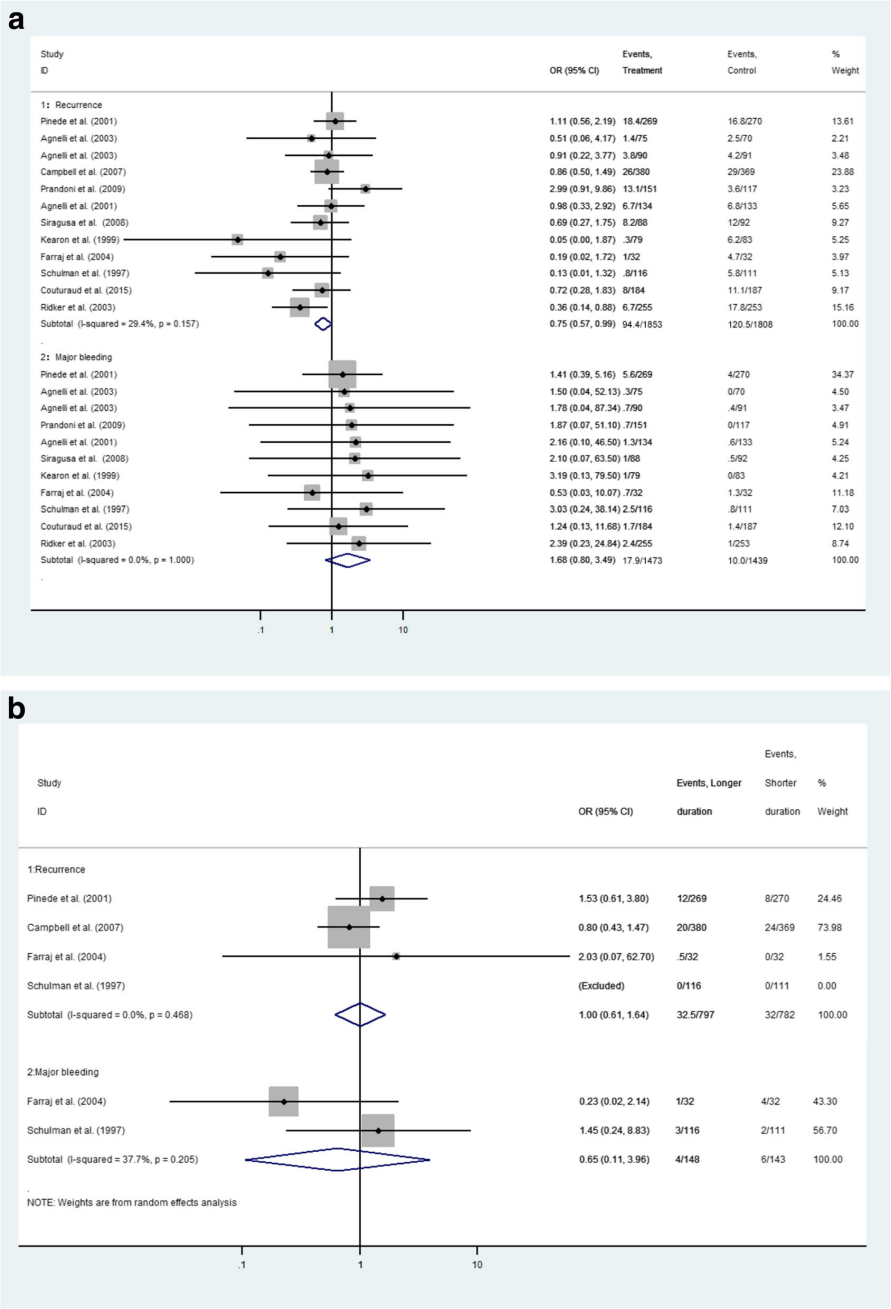
**a**-**b** Sensitivity analysis representing the risk for major bleeding after excluding single trial between longer and shorter duration for (**a**) the entire period and (**b**) during anticoagulation

Results from sensitivity analyses are reported in Fig. 2a-b. In order to investigate the reliability of the main conclusions we did the sensitivity analysis by removing one trial to exclude particular study effects. In this regard, we removed the Campbell, et.al study [12] since it was likely that it had substantially influenced the result for the major bleeding episodes. No significant differences were noted between longer and shorter duration of VKA therapy during the entire period for safety outcome (OR 1.68, 95%CI 0.80–3.49). This result was consistent with our network meta-analysis findings. Similar result was also identified during anticoagulation for major bleeding (OR 0.65, 95%CI 0.11–3.96).

## Discussion

The current network meta-analysis compares the efficacy and safety of four different durations of anticoagulation in patients with VTE or PE. With 3109 participants in 11 RCTs and an average follow-up of 33.3 months, we incorporated the evidence from these recent trials and formed the largest database on extended duration therapy of anticoagulation using VKA. The results of the present meta-analysis offered useful insights regarding the impact of extended duration of anticoagulation on clinical outcomes.

There were two main findings. First, the major findings of our meta-analysis are that longer duration (not less than 24 months) of VKA therapy is safe and associated with significant reduction in overall recurrent rate of VTE compared to short term therapy (3 months or 6 months), however, the risk of major bleeding is similar in all groups compared with each other during entire follow-up period. Second, the benefit of anticoagulation for prevention of recurrent VTE was lost after anticoagulation was discontinued and the risk of major bleeding is transiently increased at 6 months with the incidence of major bleeding decreased during the remainder of the time period of prolonged anticoagulation therapy. This meta-analysis emphasizes the need for close monitoring of the increased bleeding risk of VKA during the initial 6-month therapy duration.

Establishing the optimum duration of anticoagulation therapy of VTE is crucial for balancing the risks of recurrent VTE and bleeding complications. Consistently with previous studies evaluating extended duration of anticoagulation for prevention of recurrent VTE after a first episode of treatment, longer durations of VKA anticoagulation have reduced rates of VTE recurrence by about 70–90%, but this benefit is partially offset by a higher annual risk of major bleeding [11–21]. VKAs effectively prevent recurrent thrombosis during treatment, but they do not eliminate the risk of recurrence of VTE after discontinuing therapy and anticoagulation-related bleeding.

Based on these considerations, Current guidelines from the American College of Chest Physicians advocates at least 3 months anticoagulation therapy to prevent early recurrence [3]. Thereafter, estimations of anticipated risks of recurrent VTE and bleeding are vital to determine the optimum duration [22, 23]. In patients with isolated distal DVT or transient risk factors, anticoagulation therapy can be discontinued after 3 months; in patients with malignancy or pregnancy, low-molecular-weight heparin is preferred to VKA due to a 50% lower risk of recurrent venous thromboembolism and similar rates of major bleeding along with the teratogen concerns of VKA use in pregnancy [24–26].

However, duration of anticoagulation for unprovoked VTE remains unclear, considering extended treatment for all patients with unprovoked VTE will inevitably expose a substantial proportion of patients to an increasing risk of major bleeding. As about 50% of patients with unprovoked venous thromboembolism (VTE) will develop new episodes after discontinuing therapy, this meta-analysis suggests that indefinite treatment be considered in patients with low or moderate bleeding risk.

In light of the millions of patients treated with VKAs, a true increase in mortality associated with extended VKAs use in the management of VTE could have profound public health consequences. Conversely, widespread fear over the risks of continued VKA use could results in inappropriate discontinuation, leading to an increased risk of ischemic events. Therefore, it was imperative to examine the impact of extended treatment with VKA on mortality among VTE, to help frame trial-specific results against of the larger body of prior literature evaluating VKA duration.

The efficacy of VKA is confirmed while the major bleeding risk is the fundamental consideration for most physicians in clinical practice. To be highlighted, the side effect of taking VKA mainly occurs during the early period of medication. However, bleeding risk did not constantly increase with prolongation of VKA treatment. Our meta-analysis found that the risk of major bleeding of VKA anticoagulation treatment reached its maximum at 6 months and then decreased. The possible reasons are as follows; 1) patients’ medication compliance may have changed, with laxity in the avoidance vegetables rich in Vitamin K and frequency of obtaining an INR, and 2) patients at highest risk for VKA related bleeding may have declared themselves with a major bleeding event in the first 6 months of therapy while longer duration therapy patients are at lower risk for VKA related bleeding.

### Clinical implications

In recent years the use of direct oral anticoagulants (DOAC) is increasing in preference to prescribing VKAs for the treatment of VTE. However, VKA is cheap, effective and is available to regular monitoring and widely used in general hospitalized patients, and will remain the principal anticoagulation therapy of choice for a long time in low and middle-income countries especially for patients with liver or renal dysfunction. Additionally, it is not obvious to be aware of medication noncompliance without monitoring of DOACs. Accordingly, we perform this systematic review and meta-analysis to provide the latest and reliable evidence of randomized controlled trials, focusing on the risk of major bleeding on longer duration of VKA treatment in patients with VTE. We retrieved data both from active treatment phase and follow-up period after therapy discontinuation. This review will help clinical professionals better determine the choice of VKA treatment duration.

Our study synthesized and analyzed the existing body of evidence to identify the optimum duration that minimizes the aggregate of harm that occurs from recurrent VTE and major bleeding. By highlighting methodological issues and summarizing the results of different research studies, our study offered useful insights regarding the impact of extended anticoagulation on clinical outcomes. The risk of recurrent VTE was greatly reduced if anticoagulation was continued. If anticoagulation was discontinued in the long-term group, the risk of recurrence during the period of the study with additional follow-up was still diminished.

The present meta-analysis demonstrates that the trade-off between VTE recurrence and bleeding events with longer VKA is a matter of concern and should be carefully evaluated when the decision to continue VKA after a recommended period is considered. In current clinical practice guidelines, 3–6 months anticoagulation duration is recommended. However, according to this meta-analysis, once the 6-month major bleeding peak has passed longer anticoagulant therapy should be considered to reduce the risk of VTE recurrence. The optimal duration may vary in terms of diverse risk profiles of patients. For this respect, the decision for applying VKA should balance benefit and risk of the individual risk assessment.

### Limitations

There are some limitations in our study. A major limitation in the present study is the lack of patient-level data, resulting in difficulty to provide the time-to-event analyses. Second, heterogeneity of major bleeding and unprovoked VTE definitions are variable across RCTs. This is a potential limitation that can cause challenges to be encountered in this review. We were unable to stratify the included patients into subgroups according to different etiology (eg, patients with idiopathic venous thrombosis vs secondary venous thrombosis). In addition, other potential safety endpoints such as fatal bleeding episodes were not assessed in our review since related data cannot be extracted individually.

Third, the studies included in the meta-analysis enrolled heterogeneous populations, had differing study protocols and endpoint definitions, and compared different durations of VKA. In addition, as with any meta-analysis, our study is limited by the limitations of the included studies. The included population was not generalizable to those patients in other types of study. More studies are required to derive the optional oral VKA medication duration.

## Conclusions

In conclusion, the present meta-analysis indicates that longer duration VKA therapy was associated with significantly lower VTE recurrent rates compared with shorter duration. Although 6-month VKA treatment has the highest risk of major bleeding, if no bleeding occurs during the 6-month treatment course, the treatment duration should be extended to 24 months. An individual strategy taking into account risk of VTE recurrence, bleeding risk, therapeutic options, and patient preferences including financial considerations should be fully explored.

## Supplementary information

**Additional file 1: Table S1.** Characteristics of Included randomized controlled trials. Note: Abbreviations: pts., patients; RCT, randomized controlled trial; DB, double blind; OL, open-label; DVT, deep vein thrombosis; P-DVT, proximal deep vein thrombosis; PE, pulmonary embolism; VTE, venous thromboembolism; C-DVT, calf deep vein thrombosis; TRF, temporary risk factors; PRF, permanent risk factors; VTE, venous thromboembolism; INR, international normalized ratio, mo, month.

**Additional file 2: Table S2.** Subgroup network-meta analysis estimates of during (A) /after (B) treatment duration for VTE patients.

**Additional file 3: Figure S1.** PRISMA flow diagram of study identification for network meta-analysis. Note: PRISMA = Preferred Reporting Items for Systematic reviews and Meta-analysis; RCT = randomized controlled trials; DVT: deep thrombosis; PE: pulmonary embolism.

**Additional file 4: Figure S2.** Network of included studies with direct comparisons for VTE recurrence and major bleeding outcomes. Note: The graph represents the head-to-head Vitamin K Anticoagulants duration comparisons by connecting nodes and lines. Thickness of lines show the proportion of the number of studies comparing the two duration. Size of nodes are related to number of trials. Studies and patients are indicated by numbers above and below each line respectively.

**Additional file 5: Figure S3.** Rankings of available anticoagulation durations for treatment of VTE.

**Additional file 6: Figure S4.** Funnel plot of studies included in the meta-analysis for the risk of recurrent VTE and major bleeding.

**Additional file 7: Figure S5A-B.** Estimates of VTE recurrence and major bleeding risk between longer and shorter duration of anticoagulation in the subgroups. Note: A: During the anticoagulation B: From discontinuation to the end of follow-up.

## Data Availability

The datasets used and analyzed during the current study are available from the corresponding author on reasonable request.

## Abbreviations

DVT: Deep vein thrombosis
PE: Pulmonary embolism
RCT: Randomized controlled trial
VKA: Vitamin K antagonists
VTE: Venous thromboembolism

## References

[1] Mackman N (2008). Triggers, targets and treatments for thrombosis. Nature.

[2] Middeldorp S, Prins MH, Hutten BA. Duration of treatment with vitamin K antagonists in symptomatic venous thromboembolism. Cochrane Db Syst Rev. 2014;8:1–52. 10.1002/14651858.CD001367.pub3 PMC7074008 25092359

[3] Kearon C, Akl E, Ornelas J, Blaivas A, Jimenez D, Bounameaux H (2016). Antithrombotic therapy for VTE disease: CHEST guideline and expert panel report. Chest.

[4] Linkins LA (2013). Bleeding risks associated with vitamin K antagonists. Blood Rev.

[5] Linkins LA, Choi PT, Douketis JD (2003). Clinical impact of bleeding in patients taking oral anticoagulant therapy for venous thromboembolism - a meta-analysis. Ann Intern Med.

[6] Kearon C, Akl EA (2014). Duration of anticoagulant therapy for deep vein thrombosis and pulmonary embolism. Blood.

[7] Xu DC, Zou LL, Xing Y, Hou L, Wei YD, Zhang J (2013). Diagnostic value of ankle-brachial index in peripheral arterial disease: a meta-analysis. Can J Cardiol.

[8] Higgins JPT, Altman DG, Gotzsche PC, Juni P, Moher D, Oxman AD, et al. The Cochrane Collaboration"s tool for assessing risk of bias in randomised trials. Br Med J. 2011;343:d5928. 10.1136/bmj.d5928 PMC3196245 22008217

[9] Higgins JPT, Thompson SG, Deeks JJ, Altman DG (2003). Measuring inconsistency in meta-analyses. Brit Med J.

[10] Salanti G, Ades AE, Ioannidis JPA (2011). Graphical methods and numerical summaries for presenting results from multiple-treatment meta-analysis: an overview and tutorial. J Clin Epidemiol.

[11] Kearon C, Gent M, Hirsh J, Weitz J, Kovacs M, Anderson D (1999). A comparison of three months of anticoagulation with extended anticoagulation for a first episode of idiopathic venous thromboembolism. N Engl J Med.

[12] Campbell IA, Bentley DP, Prescott RJ, Routledge PA, Shetty HGM, Williamson IJ (2007). Anticoagulation for three versus six months in patients with deep vein thrombosis or pulmonary embolism, or both: randomised trial. Bmj.

[13] Farraj R (2004). Anticoagulation period in idiopathic venous thromboembolism. How long is enough?. Saudi Med J.

[14] Couturaud F, Sanchez O, Pernod G, Mismetti P, Jego P, Duhamel E (2015). Six months vs extended oral anticoagulation after a first episode of pulmonary embolism: the PADIS-PE randomized clinical trial. JAMA.

[15] Ridker P, Goldhaber S, Danielson E, Rosenberg Y, Eby C, Deitcher S (2003). Long-term, low-intensity warfarin therapy for the prevention of recurrent venous thromboembolism. N Engl J Med.

[16] Prandoni P, Prins MH, Lensing AW, Ghirarduzzi A, Ageno W, Imberti D, Scannapieco G (2009). Residual thrombosis on ultrasonography to guide the duration of anticoagulation in patients with deep venous thrombosis: a randomized trial. Ann Intern Med.

[17] Pinede L, Ninet J, Duhaut P, Chabaud S, Demolombe-Rague S, Durieu I (2001). Comparison of 3 and 6 months of oral anticoagulant therapy after a first episode of proximal deep vein thrombosis or pulmonary embolism and comparison of 6 and 12 weeks of therapy after isolated calf deep vein thrombosis. Circulation.

[18] Agnelli G, Prandoni P, Santamaria M, Bagatella P, Iorio A, Bazzan M (2001). Three months versus one year of oral anticoagulant therapy for idiopathic deep venous thrombosis. Warfarin optimal duration Italian trial investigators. N Engl J Med.

[19] Agnelli G, Prandoni P, Becattini C, Silingardi M, Taliani M, Miccio M (2003). Extended oral anticoagulant therapy after a first episode of pulmonary embolism. Ann Intern Med.

[20] Siragusa S, Malato A, Anastasio R, Cigna V, Milio G, Amato C (2008). Residual vein thrombosis to establish duration of anticoagulation after a first episode of deep vein thrombosis: the Duration of Anticoagulation based on Compression UltraSonography (DACUS) study. Blood.

[21] Schulman S, Granqvist S, Holmström M, Carlsson A, Lindmarker P, Nicol P (1997). The duration of oral anticoagulant therapy after a second episode of venous thromboembolism. The duration of anticoagulation trial study group. N Engl J Med.

[22] Di Nisio M, van Es N, Büller H (2016). Deep vein thrombosis and pulmonary embolism. Lancet.

[23] Wells P, Forgie M, Rodger M (2014). Treatment of venous thromboembolism. JAMA.

[24] Lee AYY, Levine MN, Baker RI, Bowden C, Kakkar AK, Prins M (2003). Low-molecular-weight heparin versus a Coumarin for the prevention of recurrent venous thromboembolism in patients with cancer. N Engl J Med.

[25] Akl E, Kahale L, Barba M, Neumann I, Labedi N, Terrenato I, et al. Anticoagulation for the long-term treatment of venous thromboembolism in patients with cancer. Cochrane Database Syst Rev. 2014(7):CD006650. 10.1002/14651858.CD006650.pub4 25004410

[26] Lee AY, Kamphuisen PW, Meyer G (2015). Tinzaparin vs warfarin for treatment of acute venous thromboembolism in patients with active cancer: a randomized clinical trial. JAMA.

