# Supplementary material for: Optimal duration of Vitamin K antagonists anticoagulant therapy after venous thromboembolism: a systematic review and network meta-analysis of randomized controlled trials
Source: BMC Cardiovasc Disord. 2020 Feb 3;20:53. doi: 10.1186/s12872-020-01345-z (PMC6998293; doi:10.1186/s12872-020-01345-z)
Supplement: Supplementary file 1 — Additional file 1: Table S1. Characteristics of Included randomized controlled trials. Note: Abbreviations: pts., patients; RCT, randomized controlled trial; DB, double blind; OL, open-label; DVT, deep vein thrombosis; P-DVT, proximal deep vein thrombosis; PE, pulmonary embolism; VTE, venous thromboembolism; C-DVT, calf deep vein thrombosis; TRF, temporary risk factors; PRF, permanent risk factors; VTE, venous thromboembolism; INR, international normalized ratio, mo, month. [file 12872_2020_1345_MOESM1_ESM.docx]

| Table S1. Characteristics of Included randomized controlled trials | | | | | | | |  | |  |  |  |  | |
| --- | --- | --- | --- | --- | --- | --- | --- | --- | --- | --- | --- | --- | --- | --- |
| **Study (Publication year)** | **Country (period)** | **Design** | **Treatment (n pts.)** | **Age (year)** | **Male**  **gender (%)** | **Follow-up (months)** | **Therapy** | | **Population** | | **Etiology** | **Episode** | **INR** |  |
|  |  |  |  |  |  |  |  |  |  |  |  |  |  |  |
| Kearon et al. 1999^[^[^11^](#_ENREF_11)^]^ | Canada (1994-1997) | RCT,  DB | 3 mo (83) | 58 ± 16 | 53.0 | 33 | warfarin | | 75% P-DVT alone 25% P-DVT + PE | | all idiopathic | a first episode of idiopathic VTE | 2.0-3.0 | |
|  |  |  | 27 mo (79) | 59 ± 16 | 68.0 | 36 |  |  |  |  |  |  |  |  |
| Agnelli et al. 2001^[^[^18^](#_ENREF_18)^]^ | Italy (1995-2000) | RCT,  OL | 3 mo (133) | 67.7 ± 7.3 | 61.2 | 37.2 | warfarin or acenocumarol | | 100% P-DVT | | all idiopathic | a first episode of idiopathic P-DVT | 2.0-3.0 | |
|  |  |  | 12 mo (134) | 66.8 ± 6.7 | 54.5 | 37.8 |  |  |  |  |  |  |  |  |
| Pinede et al. 2001^[^[^17^](#_ENREF_17)^]^ | France (1993-1999) | RCT,  OL | 3 mo (270) | > 18 | 47.6 | 15 | fluindione | | 61.3% P-DVT 34.2% C-DVT 4.5% PE alone 30% DVT+PE | | 44.6% idiopathic, 52.1% TRF, 55% PRF | a first episode of VTE | 2.0-3.0 | |
|  |  |  | 6 mo (269) | > 18 | 47.0 | 15 |  |  |  |  |  |  |  |  |
| Agnelli et al. 2003^[^[^19^](#_ENREF_19)^]^ | Italy (1997-2000) | RCT,  OL | 3 mo (70) | 61.0 ± 15.5 | 41.6 | 33.9 | warfarin or acenocumarol | | 100% PE | | 100% TRF | a first episode of PE | 2.0-3.0 | |
|  |  |  | 6 mo (75) | 62.9 ± 16.3 | 39.4 | 35 |  |  |  |  |  |  |  |  |
|  |  |  | 3 mo (91) | 61.0 ± 15.5 | 41.6 | 31.7 |  |  | 100% PE | | all idiopathic | a first episode of PE |  |  |
|  |  |  | 12 mo (90) | 62.9 ± 16.3 | 39.4 | 34.8 |  |  |  |  |  |  |  |  |
| Campbell et al. 2007^[^[^22^](#_ENREF_22)^]^ | UK  (1999-2002) | RCT | 3 mo (369) | 59.0 ± 15.7 | 50.0 | 12 | warfarin | | 70% DVT alone 30% DVT+PE | | all idiopathic | Unclear | 2.0-3.5 | |
|  |  |  | 6 mo (380) | 58.5 ± 15.0 | 57.0 | 12 |  |  |  |  |  |  |  |  |
| Farraj et al. 2004^[^[^13^](#_ENREF_13)^]^ | Jordan (1998-2001) | RCT | 6 mo (32) | 42 ± 14 | 56.3 | 18 | warfarin | | VTE | | all idiopathic | a first episode of idiopathic VTE | 2.0-3.0 | |
|  |  |  | 24 mo (32) | 41 ± 15 | 62.5 | 36 |  |  |  |  |  |  |  |  |
| Schulman et al. 1997^[^[^21^](#_ENREF_21)^]^ | Sweden (1988-1991) | RCT, OL | 6 mo (111) | 65 ± 12.4 | 63.0 | 48 | warfarin or dicumarol | | 85% DVT 15% DVT+PE | | 19% TRF | a second episode of VTE | 2.0-2.85 | |
|  |  |  | indefinite (116) | 64 ± 12.5 | 59.0 | 48 |  |  |  |  |  |  |  |  |
| Couturaud et al. 2015^[^[^23^](#_ENREF_23)^]^ | France (2007-2014) | RCT, DB | 6 mo (187) | 57.3 ± 17.4 | 55.1 | 42 | warfarin | | 100% PE | | symptomatic unprovoked PE | a first episode of unprovoked PE | 2.0-3.0 | |
|  |  |  | 24 mo (184) | 58.7 ± 17.9 | 42.4 | 42 |  |  |  |  |  |  |  |  |
| Ridker et al. 2003^[^[^15^](#_ENREF_15)^]^ | The United States  (1998-2002) | RCT, DB | 6.5 mo (253) | 53 | 52.6 | 25 | warfarin | | VTE | | 62% idiopathic 38%, nonidiopathic PRF | Unclear | 1.5-2.0 | |
|  |  |  | 31.5 mo (255) | 53 | 52.9 | 25 |  |  |  |  |  |  |  |  |
| Prandoni et al. 2009^[^[^16^](#_ENREF_16)^]^ | Italy (1999-2003) | RCT, OL | 3 mo (117) | 58(23-92) | 41 | 33 | warfarin | | 100% P-DVT | | 56.3% idiopathic, 43.7% secondary | a first episode of acute P-DVT | 2.0-3.0 | |
|  |  |  | 6 mo (151) | 69(21-89) | 57.6 | 33 |  |  |  |  |  |  |  |  |
| Siragusa et al. 2008^[^[^20^](#_ENREF_20)^]^ | Italy (2005-2007) | RCT, OL | 3 mo (92) | 61.1 ± 11.5 | 52.3 | 25 | warfarin or acenocumarol | | 100% P-DVT | | 76.7% idiopathic, 23.3% provoked | a first episode of P-DVT | 2.0-3.0 | |
|  |  |  | 12 mo (88) | 57.1 ± 14.1 | 53.3 | 25 |  |  |  |  |  |  |  |  |

Abbreviations: pts., patients; RCT, randomized controlled trial; DB, double blind; OL, open-label; DVT, deep vein thrombosis; P-DVT, proximal deep vein thrombosis; PE, pulmonary embolism; VTE, venous thromboembolism; C-DVT, calf deep vein thrombosis; TRF, temporary risk factors; PRF, permanent risk factors; VTE, venous thromboembolism; INR, international normalized ratio, mo, month.
