# Supplementary material for: Optimal duration of Vitamin K antagonists anticoagulant therapy after venous thromboembolism: a systematic review and network meta-analysis of randomized controlled trials
Source: BMC Cardiovasc Disord. 2020 Feb 3;20:53. doi: 10.1186/s12872-020-01345-z (PMC6998293; doi:10.1186/s12872-020-01345-z)
Supplement: Supplementary file 2 — Additional file 2: Table S2. Subgroup network-meta analysis estimates of during (A) /after (B) treatment duration for VTE patients. [file 12872_2020_1345_MOESM2_ESM.docx]

Table S2. Subgroup network-meta analysis estimates of during (A)/after (B) treatment duration for VTE patients

(A)

|  | Odds ratio (95% Confidence Interval) for outcome of major bleeding | | | |
| --- | --- | --- | --- | --- |
| Odds ratio (95% Confidence Interval) for outcome of VTE recurrence | A (3 months) | 33.45 (2.00, 559.67)^*^ | - | 22.11 (0.94, 518.68) |
|  | **0.98 (0.59, 1.62)** | B (6 months) | - | 0.66 (0.16, 2.74) |
|  | **-** | **-** | C (12 months) | - |
|  | **1.43 (0.10, 19.95)** | **1.47 (0.11, 19.43)** | **-** | D (≥24 months) |

(B)

|  | Odds ratio (95% Confidence Interval) for outcome of major bleeding | | | |
| --- | --- | --- | --- | --- |
| Odds ratio (95% Confidence Interval) for outcome of VTE recurrence | A (3 months) | 1.12 (0.07, 17.95) | 0.45 (0.02, 12.82) | 0.55 (0.02, 14.31) |
|  | **1.04 (0.70, 1.55)** | B (6 months) | 0.40 (0.01, 31.15) | 0.49 (0.09, 2.73) |
|  | **1.04 (0.35, 3.06)** | **0.99 (0.31, 3.14)** | C (12 months) | 1.22 (0.01, 130.98) |
|  | **0.69 (0.33, 1.45)** | **0.66 (0.35, 1.25)** | **0.67(0.18, 2.46)** | D (≥24 months) |

Note: The during (A) /after treatment (B) results from network meta-analysis are presented as odds ratio (95% Confidence Intervals) between the column-defining and row-defining treatment duration. Odds ratio for comparisons are in the cell in common between the column-defining and row-defining treatment. For outcome of VTE recurrence, row treatment is compared with column treatment (ie, column treatment is reference). For outcome of major bleeding, column treatment is compared with row treatment (ie, row treatment is reference). Numbers in brackets indicate 95% confidence intervals. Data in bold represents the primary efficacy outcome (VTE recurrence) and the rest represents the primary safety outcome (major bleeding). VTE, venous thromboembolism.

A-D: Treatment duration.

*: Statistically significant results.

-: No available data was used in subgroup analysis.
