# Supplementary figures and images for: Optimal duration of Vitamin K antagonists anticoagulant therapy after venous thromboembolism: a systematic review and network meta-analysis of randomized controlled trials
Source: BMC Cardiovasc Disord. 2020 Feb 3;20:53. doi: 10.1186/s12872-020-01345-z (PMC6998293; doi:10.1186/s12872-020-01345-z)

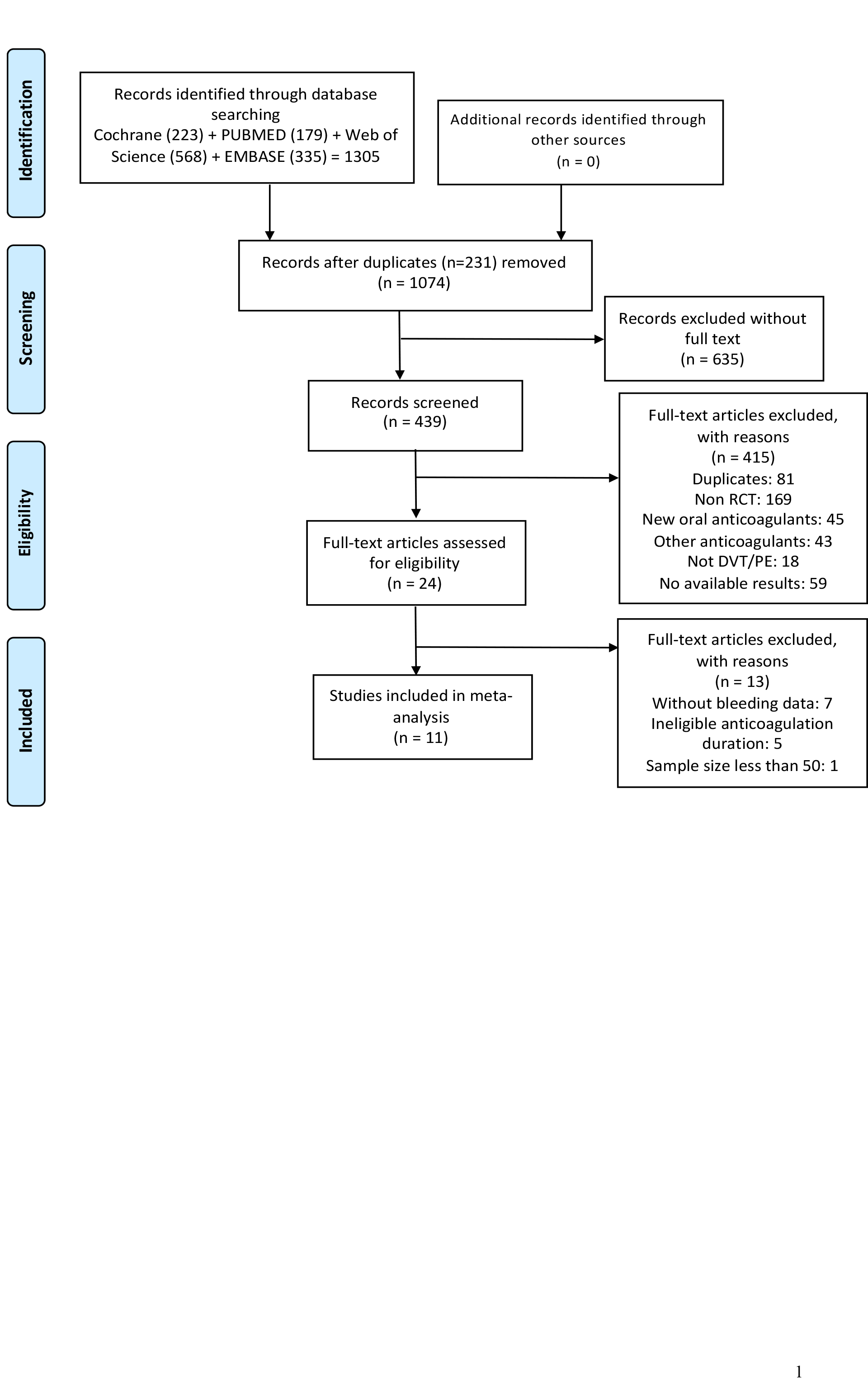

Supplement: Supplementary file 3 — Additional file 3: Figure S1. PRISMA flow diagram of study identification for network meta-analysis. Note: PRISMA = Preferred Reporting Items for Systematic reviews and Meta-analysis; RCT = randomized controlled trials; DVT: deep thrombosis; PE: pulmonary embolism. [file 12872_2020_1345_MOESM3_ESM.tif]

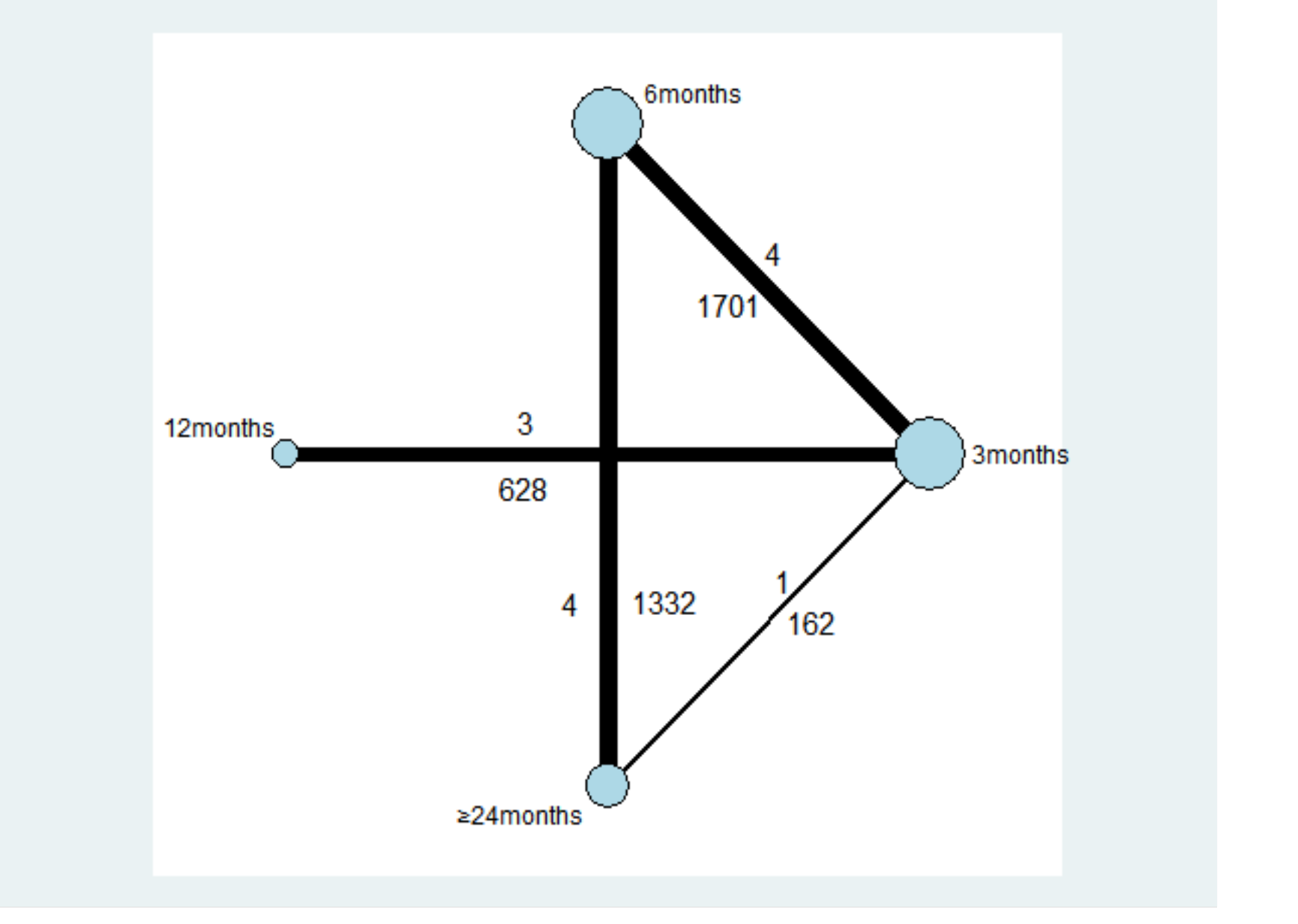

Supplement: Supplementary file 4 — Additional file 4: Figure S2. Network of included studies with direct comparisons for VTE recurrence and major bleeding outcomes. Note: The graph represents the head-to-head Vitamin K Anticoagulants duration comparisons by connecting nodes and lines. Thickness of lines show the proportion of the number of studies comparing the two duration. Size of nodes are related to number of trials. Studies and patients are indicated by numbers above and below each line respectively. [file 12872_2020_1345_MOESM4_ESM.tif]

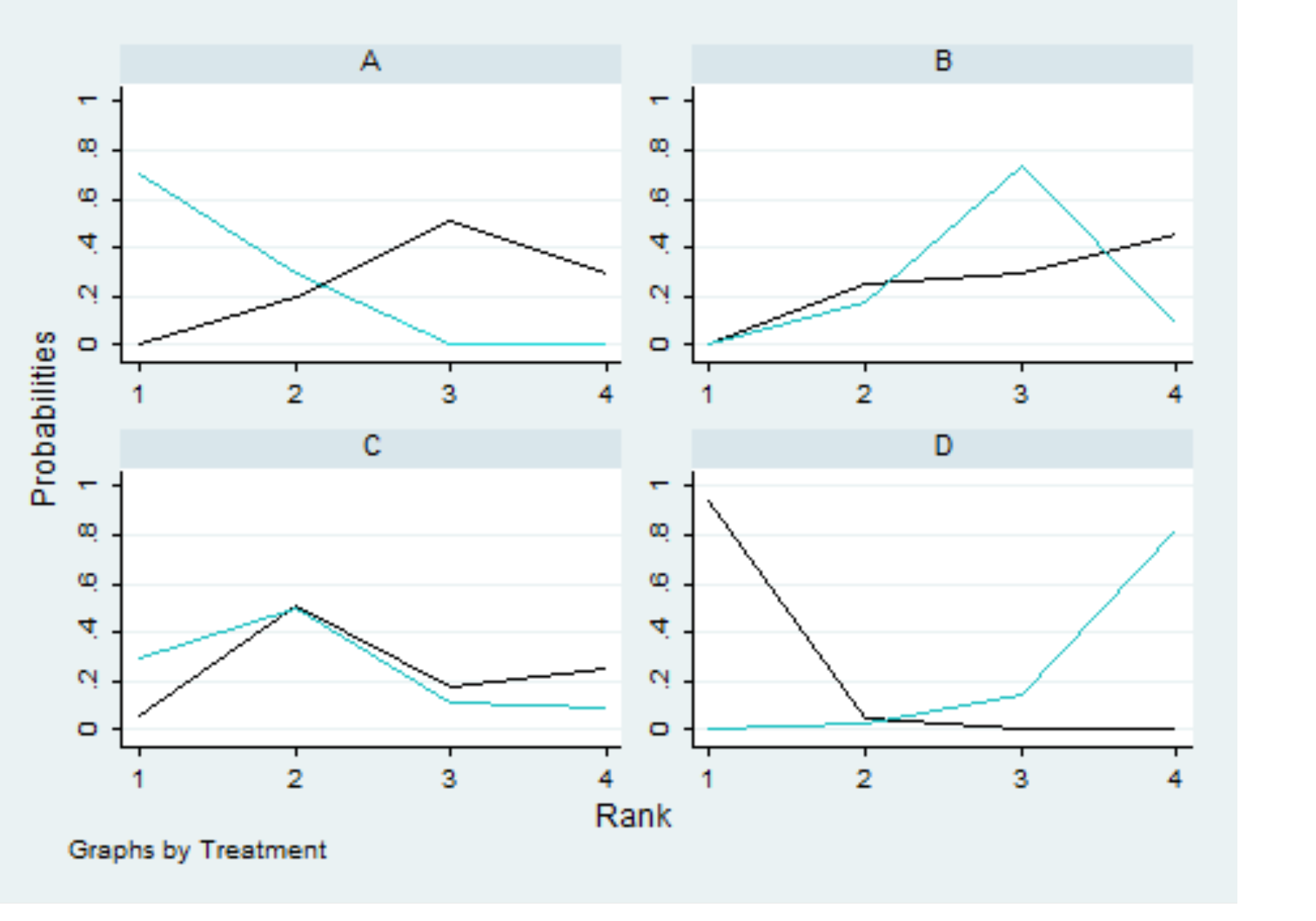

Supplement: Supplementary file 5 — Additional file 5: Figure S3. Rankings of available anticoagulation durations for treatment of VTE. [file 12872_2020_1345_MOESM5_ESM.tif]

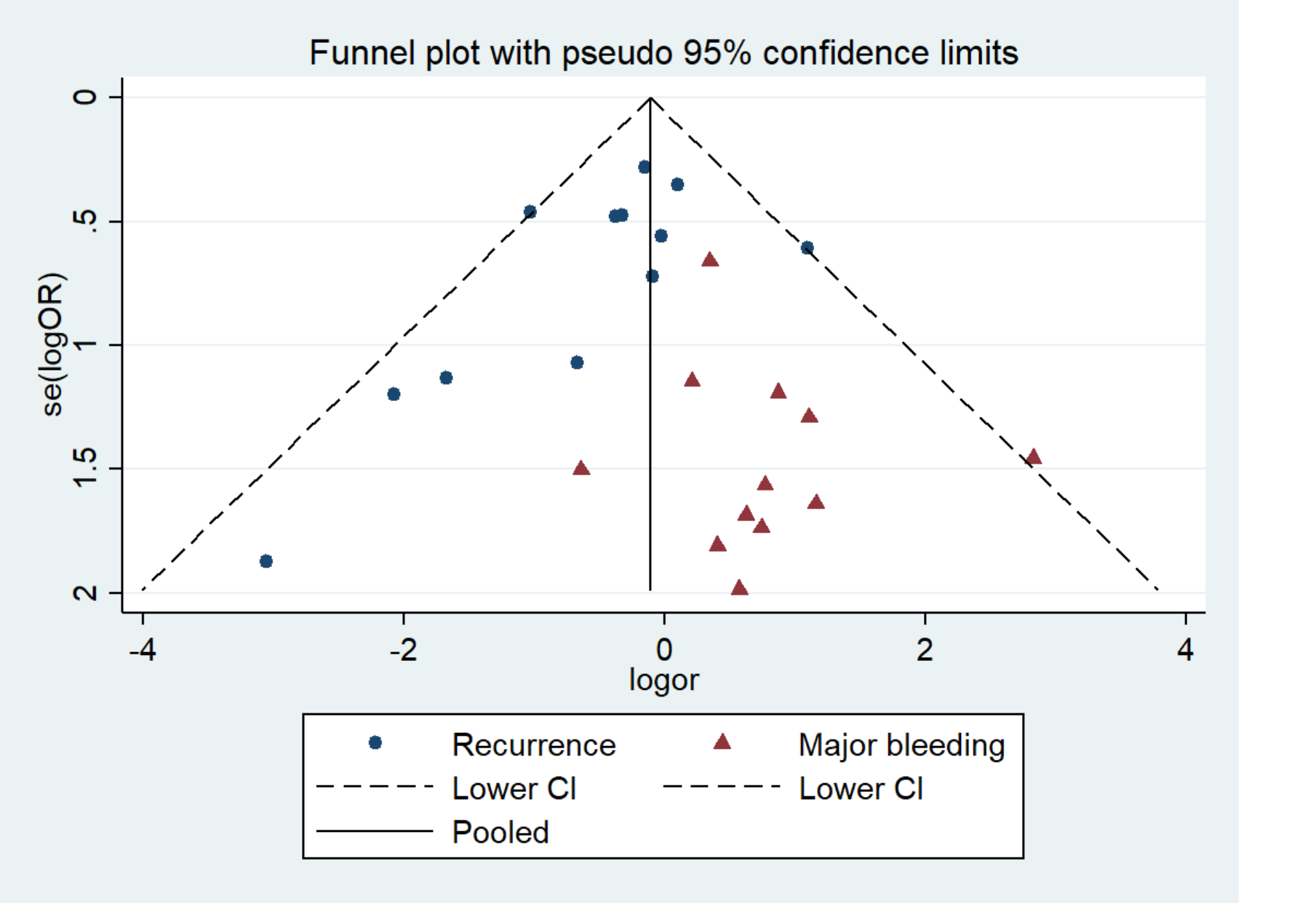

Supplement: Supplementary file 6 — Additional file 6: Figure S4. Funnel plot of studies included in the meta-analysis for the risk of recurrent VTE and major bleeding. [file 12872_2020_1345_MOESM6_ESM.tif]

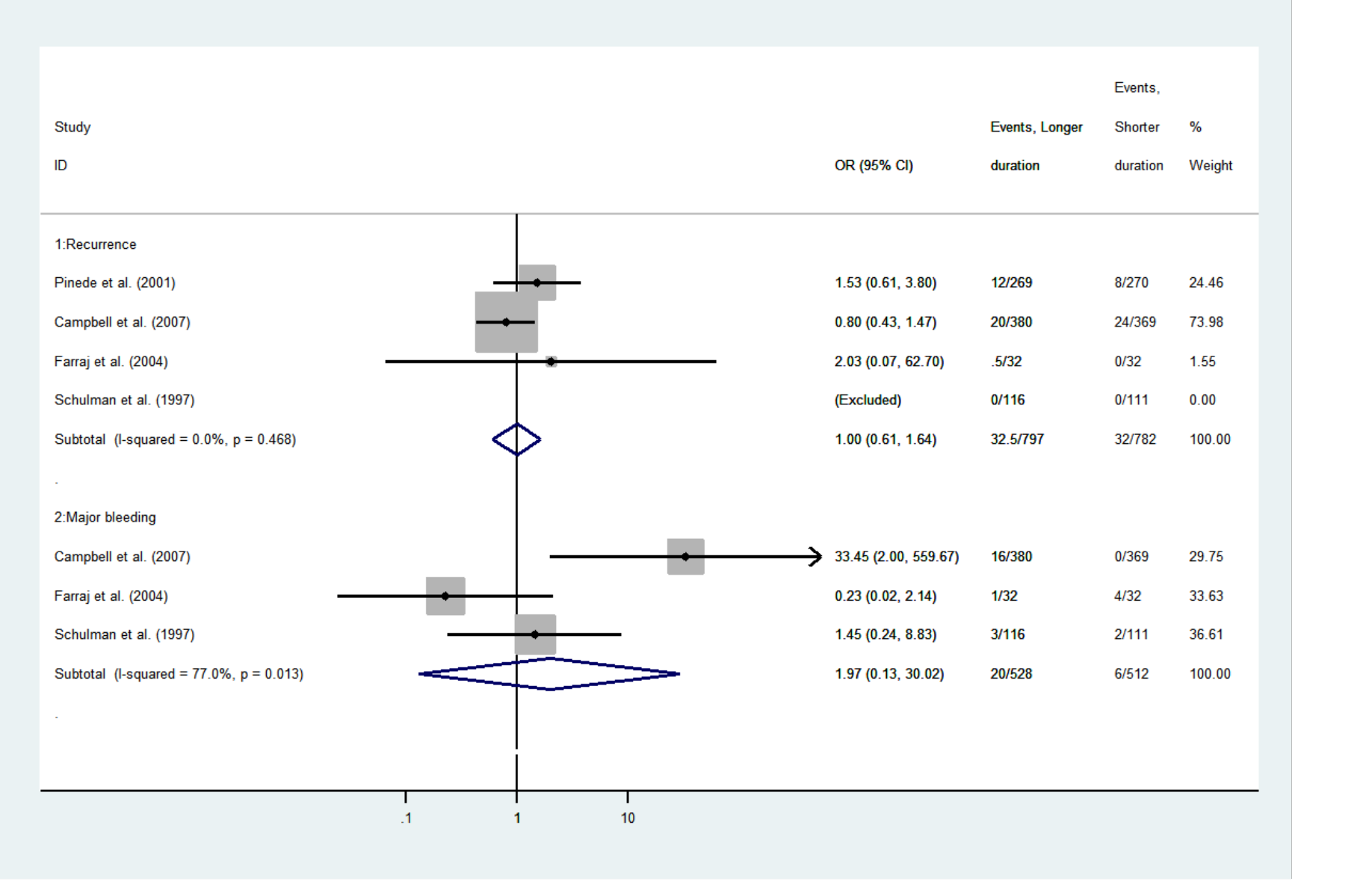

Supplement: Supplementary file 7 — Additional file 7: Figure S5A-B. Estimates of VTE recurrence and major bleeding risk between longer and shorter duration of anticoagulation in the subgroups. Note: A: During the anticoagulation B: From discontinuation to the end of follow-up. [file 12872_2020_1345_MOESM7_ESM.zip › Additional file 5-Figure S5AR5.tif]

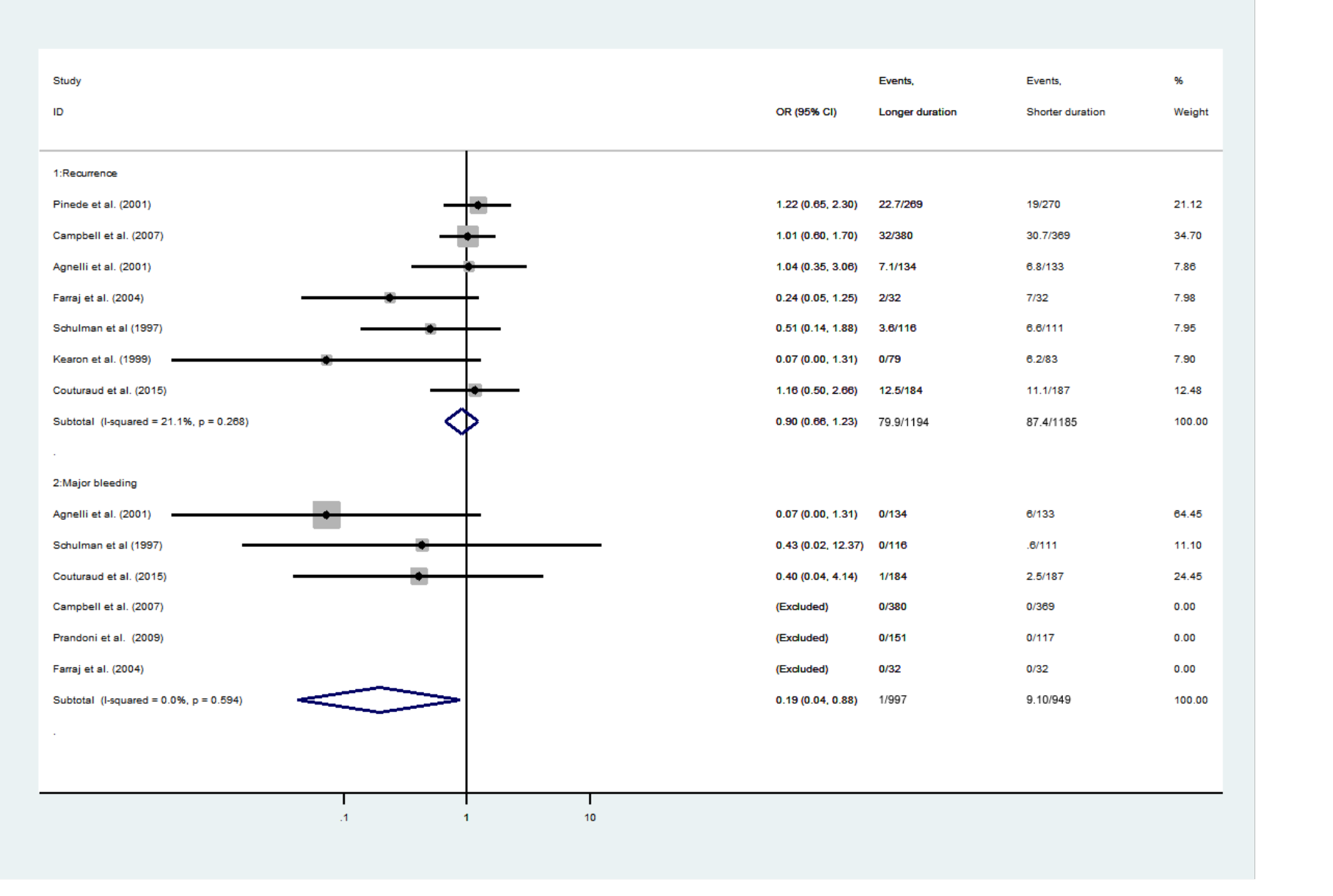

Supplement: Supplementary file 7 — Additional file 7: Figure S5A-B. Estimates of VTE recurrence and major bleeding risk between longer and shorter duration of anticoagulation in the subgroups. Note: A: During the anticoagulation B: From discontinuation to the end of follow-up. [file 12872_2020_1345_MOESM7_ESM.zip › Addtional file 6-Figure S5BR5.tif]
